# Assessment of body composition, quality of life, and depression in women with polycystic ovary syndrome

**DOI:** 10.20945/2359-4292-2026-0036

**Published:** 2026-04-01

**Authors:** Thalita Ponce, Patrícia dos Santos Vigário, Cloyra de Paiva Almeida, Flávia Lucia Conceição

**Affiliations:** 1 Programa de Pós-graduação em Endocrinologia, Universidade Federal do Rio de Janeiro, Rio de Janeiro, RJ, Brasil; 2 Programa de Pós-graduação em Ciências da Reabilitação, Programa de Pós-graduação em Desenvolvimento Local, Centro Universitário Augusto Motta, Rio de Janeiro, RJ, Brasil; 3 Instituto de Psiquiatria da Universidade Federal do Rio de Janeiro, Rio de Janeiro, RJ, Brasil

**Keywords:** Polycystic ovary syndrome, body composition, depression, quality of life, obesity.

## Abstract

**Objective:**

This study aimed to evaluate body composition, quality of life, and depression risk in women with polycystic ovary syndrome (PCOS) compared to women without PCOS.

**Subjects and methods:**

This prospective, cross-sectional study assessed quality of life (QoL) and depressive symptoms in women with polycystic ovary syndrome (PCOS) compared with controls with and without overweight or obesity. Assessments included body composition, QoL, health-related quality of life (HRQoL), and depressive symptoms. Participants without PCOS were divided into two groups according to BMI: < 25 kg/m^2^ [median age 30 years (27-33)] and ≥ 25 kg/m^2^ [median age 32 years (29-35)].

**Results:**

The study included 47 women with polycystic ovary syndrome (PCOS) phenotypes A and B. The median age was 29 years (25-35), and the median body mass index (BMI) was 32.08 kg/m^2^ (28.48-36.40). Women with PCOS showed a higher risk of depression (24% with moderate to severe risk) compared with women without PCOS, both with and without overweight or obesity (5% and 3%, respectively). Additionally, women with PCOS consistently reported lower QoL and HRQoL scores, particularly in the physical, environmental, and overall QoL domains, as well as in functional capacity, pain, general health status, vitality, emotional well-being, and mental health domains of HRQoL.

**Conclusion:**

Based on these findings, we concluded that body composition does not appear to be a determining factor for increased risk of depressive symptoms or poorer perceptions of QoL and HRQoL in women with PCOS.

## INTRODUCTION

Polycystic ovary syndrome (PCOS) is the most common endocrine disorder among women of reproductive age, affecting approximately 10%-13% of this population. The syndrome exhibits a complex etiology involving reproductive, metabolic, and psychological factors, and often remains undiagnosed until later stages ^(1)^. Although its exact cause is not fully understood ^(2)^, multiple factors are believed to contribute to the development of PCOS, including genetic predisposition ^(2)^, metabolic disturbances ^(3)^, behavioral factors ^(4)^, hormonal alterations ^(5)^, and overweight or obesity ^(6)^. According to the 2003 Rotterdam Consensus ^(7)^, PCOS is diagnosed when at least two of the following three criteria are present: clinical and/or biochemical hyperandrogenism (HA), oligo/anovulation (AO), and polycystic ovary morphology (POM). Patients may be categorized into one of four phenotypes: A) HA + AO + POM, B) HA + AO, C) HA + POM, and D) AO + POM ^(8)^.

PCOS is associated with an elevated risk of various metabolic conditions, including insulin resistance, type 2 diabetes mellitus, hypertension, and dyslipidemia. Psychological disorders are also among the comorbidities linked to the syndrome ^(9)^. In a 2023 narrative review, the authors highlighted the bidirectional relationship between metabolic syndrome and major depressive disorders, demonstrating that heightened activity of pro-inflammatory enzymes, combined with a diminished response of anti-inflammatory enzymes - both common in metabolic diseases - can result in dysfunctional metabolism and neurodegeneration ^(10)^. These effects, whether occurring individually or collectively, can significantly impair quality of life (QoL).

In its position statement, the European Society of Endocrinology (Gerard and cols., 2014) recommends that psychological assessment for patients with PCOS should be conducted not only at initial evaluation, but as an integral part of ongoing care ^(11)^. The 2023 International Guide to the Evaluation and Management of PCOS further emphasizes the importance of assessing the increased risks of anxiety and depression in women diagnosed with PCOS, advocating for continuous psychological monitoring in this population ^(12)^.

Nevertheless, it remains unclear which factors most strongly contribute to the lower perceived QoL and higher prevalence of depressive symptoms observed in women with PCOS. Overweight and obesity, which are common in PCOS, are significantly linked to these emotional outcomes ^(13)^. Additionally, metabolic and reproductive complications, as well as physical manifestations such as hirsutism, hair loss, and acne, may also play a role ^(14)^.

In this context, we aimed to assess signs and symptoms of depression, perceived QoL, and health-related QoL (HRQoL) in women with PCOS classified as phenotypes A and B, and to compare them with two control groups of women without PCOS, both with and without overweight or obesity. The hypothesis was that PCOS is associated with poorer perceptions of QoL and HRQoL, regardless of overweight or obesity status. Therefore, for practical application during the diagnostic process, it is important to evaluate emotional health, refer patients for psychological assessment, and promote comprehensive treatment to achieve better outcomes.

## SUBJECTS AND METHODS

### Ethical considerations

This study was approved by the Institutional Ethics Committee (no. 04057312.6.0000.525). All participants received clear and objective information about the study and signed an informed consent form.

### Study design and sample

This prospective cross-sectional study included 47 women diagnosed with PCOS phenotypes “A” and “B” (median age: 29 years [interquartile range - IQR: 25-35]; median body mass index [BMI]: 32.08 kg/m^2^ [IQR: 28.48-36.40]). Two control groups without PCOS were also assessed: the first comprised 47 women with BMI < 25 kg/m^2^ (median age: 30 years [IQR: 27-33]; median BMI: 22 kg/m^2^ [IQR: 20.55-23.15]) (control BMI < 25 kg/m^2)^, and the second comprised 32 women with BMI ≥ 25 kg/m^2^ (median age: 32 years [IQR: 29-35]; median BMI: 28.6 kg/m^2^ [IQR: 26.6-31.00]) (control BMI ≥ 25 kg/m^2)^. Participants with PCOS were recruited from the Endocrinology outpatient clinics at Clementino Fraga Filho University Hospital (Federal University of Rio de Janeiro). Control participants were recruited among female students and staff at the Federal University of Rio de Janeiro, with no history of PCOS or other chronic diseases, and were divided into two groups based on BMI.

Sample size calculations were performed with Epi Info 6.0 software (StartCalc - Sample Size and Power command), assuming an 80% statistical power, a significance level of 5% (*p* < 0.05), and a population prevalence of PCOS between 8%-10%. For continuous variables, the minimum detectable difference between groups was estimated according to prior studies, with the expected standard deviation reflective of the study population. For categorical variables, the expected prevalence and an acceptable margin of error were considered. Assuming a study power of 99% and a confidence level of 95% (*p* = 0.05), an estimated sample size of 60 participants was determined, with 20 in the PCOS group and 20 controls in each subgroup. The final sample size was increased to account for potential dropouts, ensuring statistical robustness.

Inclusion criteria for women with PCOS were: age 18-45 years, medical records at the university hospital, and fulfillment of at least two of the three Rotterdam Consensus criteria for PCOS diagnosis ^(7)^, with exclusion of other causes of hyperandrogenism. Eligible women were evaluated during clinical consultations and invited to participate in the study. Women with chronic diseases, such as diebtes mellitus, dyslipidemia, hypertension, or hypothyroidism, were excluded. Clementino Fraga Filho University Hospital is a tertiary-level university hospital providing specialized, high-complexity care. As a result, patients treated at this institution often present with more severe cases of PCOS, likely explaining why only phenotypes A and B were observed in the sample.

### Procedures

Women with PCOS registered at the outpatient clinic were contacted by the study coordinator, informed about the study, and scheduled for a physician consultation. After verification of diagnosis and eligibility, they received detailed information about the study and, upon agreeing to participate, completed and signed the informed consent form. Women with PCOS then underwent anamnesis during a clinical consultation with an endocrinologist participating in the study. Demographic data were collected, and patients were asked about amenorrhea, hirsutism, acne, anovulation, baldness, and weight gain. In addition to these signs and symptoms, patients were questioned regarding diabetes, systemic arterial hypertension, family history of PCOS or metabolic diseases, and cardiovascular diseases. Following anamnesis, a comprehensive physical examination was performed to assess hirsutism, acne, and acanthosis nigricans.

At the end of the consultation, patients underwent body composition assessment and completed three questionnaires: the WHOQOL-Bref for perceived QoL ^(15)^, the Medical Outcomes Study 36-Item Short-Form Health Survey (SF-36) for HRQoL ^(16)^, and the Beck Depression Inventory for assessment of depressive symptoms ^(17)^. All questionnaires had been translated and validated in Portuguese. Data collected from women with PCOS were analyzed and compared with those from two non-PCOS control groups defined according to BMI: < 25 kg/m^2^ and ≥ 25 kg/m^2^. The control group with BMI ≥ 25 kg/m^2^ was not matched to the PCOS group because the PCOS group consisted predominantly of individuals with obesity, whereas the control group was mainly composed of women with overweight.

### Body composition

Body composition was estimated using standard anthropometric methods. Total body mass (kg) was measured with an electronic scale (Filizola; precision: 0.1 kg), and height (cm) was measured with a stadiometer (Filizola; precision: 0.1 cm). Seven skinfold thicknesses were measured in millimeters using a skinfold caliper (CESCORF; precision: 1 mm) at the abdominal, suprailiac, subscapular, pectoral, mid-axillary, triceps, and thigh sites. Waist, abdominal, and hip circumferences (cm) were measured with a flexible metal tape (Cardiomed; precision: 1 mm). Bone diameters (femur and humerus bi-epicondylar, and bi-styloid) were measured in centimeters with a pachymeter (Cardiomed; precision: 1 mm). From these measurements, the following variables were calculated: body mass index (BMI; kg/m^2)^ ^(18)^, body density ^(19)^, fat percentage (%F) ^(20)^, fat mass, lean mass, and muscle mass ^(21)^.

The Jackson and Pollock 7-site skinfold protocol for women, widely validated in the literature, was utilized. As DEXA, the gold standard for body composition assessment, was unavailable during the study period, a validated method appropriate for the studied age group was chosen, thus ensuring reliable and comparable results. All measurements were performed in accordance with International Society for the Advancement of Kinanthropometry standards ^(22)^, and always by the same assessor, who had prior experience in anthropometric assessment. The assessor demonstrated excellent reliability, with intraclass correlation coefficients ranging from 0.978 to 0.998 for the seven skinfold sites (i.e., abdominal, suprailiac, subscapular, pectoral, mid-axillary, triceps, and thigh).

### Signals and symptoms of depression

To assess depressive symptoms, the Beck Depression Inventory (BDI), validated in Portuguese, was administered. The BDI covers items related to sadness, pessimism, feelings of failure, guilt and punishment, dissatisfaction, self-deprecation, self-accusation, suicidal ideation, crying episodes, irritability, social withdrawal, decision-making difficulties, distorted self-image, work inhibition, sleep disturbances, fatigue, appetite and weight loss, worry, and decreased libido ^(17)^. BDI scores are classified as follows for individuals with no prior history of psychological disorders: up to 10 points, absent or minimal symptoms; 10-18 points, mild depression; 19-29 points, moderate to severe depression; and 30-63 points, severe depression ^(23)^.

### Quality of life and health-related quality of life

The self-administered WHOQOL-Bref questionnaire, translated and validated in Portuguese, was used to assess QoL. It contains 26 questions divided into four domains: physical health, psychological, social relationships, and environment, two general QoL questions. Patients were instructed to answer based on their experiences over the prior fifteen days. Responses were provided on Likert-type scales, with higher scores indicating more positive perceptions, except for questions 3, 4, and 26, which are reverse-scored ^(15)^. The self-administered SF-36 questionnaire, also translated and validated in Portuguese, was applied to assess HRQoL ^(16)^. This instrument comprises 36 items on a Likert-type scale, covering eight domains: functional capacity, physical functioning, pain, general health, vitality, social functioning, emotional aspects, and mental health. Each domain yields an independent score (0-100), with higher scores reflecting better perceived HRQoL.

### Statistical analysis

The Kolmogorov-Smirnov test was used to assess the distribution of variables. Parametric data were analyzed using one-way analysis of variance with Bonferroni post hoc test. Non-parametric data were analyzed with the Kruskal-Wallis test followed by Dunn’s post hoc test. For assessment of depression risk, a frequency graph was constructed to quantify the proportion of scores in the categories: below 10, 10-18, and 19-29, across the three groups. Subsequently, Spearman’s correlation test was applied to explore relationships between body composition and depression risk. A *p*-value less than 0.05 was considered statistically significant. All analyses were conducted using GraphPad Prism 8.0 software.

## RESULTS

**Figure 1:**
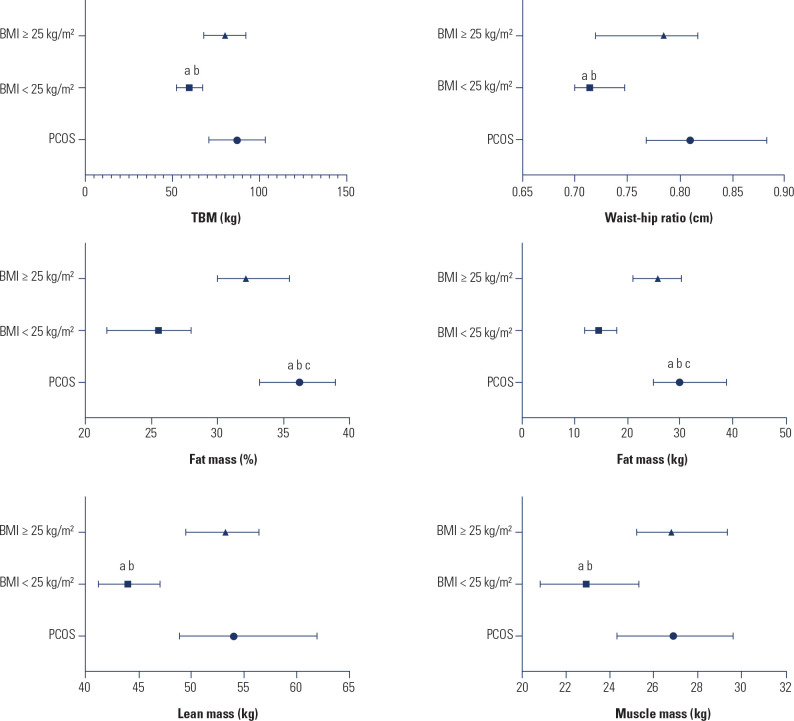
Body composition. Data are presented as median [first quartile - third quartile]; TBM = total body mass; statistical significance *p* < 0.05; ^a^difference between PCOS and control BMI < 25 kg/m^2^; ^b^difference between control BMI < 25 kg/m^2^ and control BMI ≥ 25 kg/m^2^; ^c^difference between PCOS and control BMI ≥ 25 kg/m^2^.

After clinical and laboratory evaluation, all patients exhibited hyperandrogenism. The primary complaints were oligo/anovulation (35.3%), assessed based on menstrual irregularities rather than second-phase progesterone levels, as it was not feasible to assess all patients; hirsutism, determined using the Ferriman-Gallwey scale with a cutoff point of 8 (the threshold used at the onset of data collection) (35.3%), acne (25.5%), infertility (23.5%), and baldness (7.9%). These characteristics confirm that our cohort corresponds to phenotypes A and B, classified as classic PCOS. Body composition was assessed by a physical education professional, who found that all women with PCOS were overweight or obese (**Figure 1**).

Of the 47 women evaluated, 32 adhered to the treatment protocol. During follow-up, we investigated risk indicators for metabolic syndrome, with the most prevalent being fasting glucose > 126 mg/dL, HDL cholesterol < 50 mg/dL, and abdominal circumference > 88 cm. **Table 1** presents the number and percentage of patients displaying one, two, or three metabolic syndrome risk indicators.

**Table 1:** Prevalence of metabolic syndrome risk factors in the study population

	3 or more MS risk factors	2 MS risk factors	1 MS risk factors
Number of patients	11 patients	12 patients	9 patients
Percentage of patients	34%	38%	28%

MS: metabolic syndrome.

**Figure 2:**
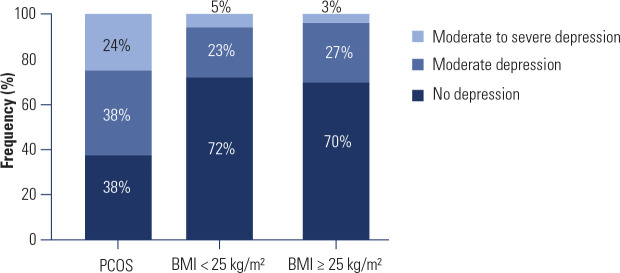
Beck Depression Inventory - percentage of women in the different groups based on risk of depression.

Frequency analysis (**Figure 2**) demonstrated that 24% of women with PCOS exhibited a moderate to severe risk of depression, compared to only 5% in the BMI > 25 control group and 3% in the BMI < 25 control group. Additionally, 38% of women with PCOS were classified as having a moderate risk of depression, whereas this proportion was 27% and 23% in the BMI > 25 and BMI < 25 control groups, respectively. Conversely, 38% of women with PCOS were classified as having no depression, in comparison to 72% and 70% in the BMI < 25 and BMI > 25 control groups, respectively. To assess the statistical difference in the incidence of depressive symptoms between groups, a Chi-square test was performed (χ^2^ = 15.54; *p* = 0.0037), indicating a statistically significant difference in depression symptom incidence among groups.

**Figure 3:**
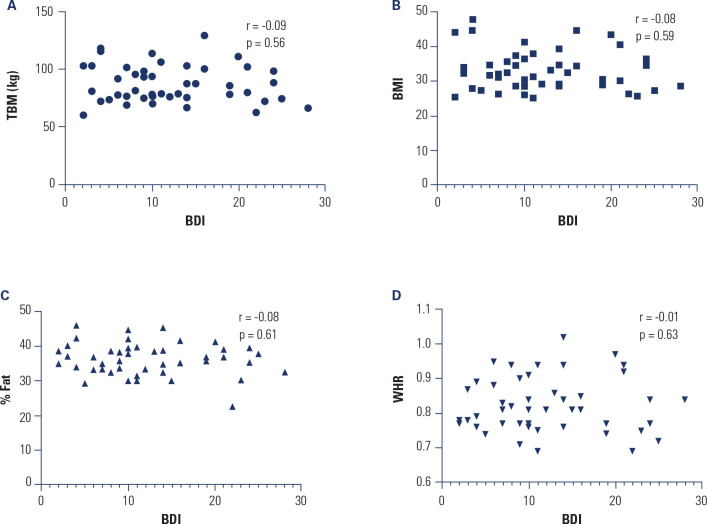
Body composition vs. symptoms of depression - correlation analysis between body composition data and risk of depression.

BDI: beck depression inventory; TBM: total body mass; BMI: body mass index; % Fat: body fat percentage; WHR: waist-to-hip ratio.

Spearmans correlation analysis revealed no significant associations between depression risk and body composition parameters in women with PCOS: total body mass (*r* = 0.09; *p* = 0.56), BMI (*r* = 0.08; *p* = 0.59), fat percentage (%G) (*r* = 0.08; *p* = 0.61), and waist-to-hip ratio (WHR) (*r* = 0.01; *p* = 0.93) (**Figure 3**).

Regarding QoL, as measured by WHOQOL-Bref, statistically significant differences were observed in the physical (*p* < 0.0011), environmental (*p* < 0.0001), and overall QoL (*p* < 0.0014) domains. Specifically, in the physical domain, the control group with BMI ≥ 25 kg/m^2^ reported significantly better perceptions than both women with PCOS and controls with BMI < 25 kg/m^2^. In the environmental domain, controls with BMI < 25 kg/m^2^ exhibited better perceptions, with statistically significant differences compared to women with PCOS and controls with BMI ≥ 25 kg/m^2^. In the overall QoL domain, women with PCOS had worse perceptions than the control group with BMI < 25 kg/m^2^, with statistically significant differences (**Table 2**).

**Table 2:** WHOQOL-Bref domain scores according to study subgroups

	PCOS (n=47)	Control BMI < 25 kg/m^2^(n=47)	Control BM I ≥ 25 kg/m^2^ (n=30)	^*^P-value
Physical	13.10^bc^[10.30-17.70]	13.10 [8.0-18.30]	15.15 [9.70-19.40]	<0.0011
Emotional	13.30 [9.30-17.30]	14.0 [9.30-18.0]	14.70 [9.30-17.30]	<0.0504
Social relations	14.70 [6.70-20.0]	14.00 [9.30-18.00]	16.00 [8.00-20.00]	<0.0513
Environmental	13.00^ab^[5.0-18.0]	16.00 [9.30-20.0]	13.50 [9.50-16.50]	<0.0001
General QoL issues	12.00^a^[4.0-18.0]	14.00 [8.00-20.00]	14.00 [8.00-18.00]	<0.0014

Values described as median [minimum value - maximum value]; QoL = quality of life;

* Kruskal-Wallis; statistical significance = 0.016%;

a difference between PCOS and control BMI < 25 kg/m^2^ by Mann-Whitney;

b difference between control BMI < 25 kg/m^2^ and control BMI ≥ 25 kg/m^2^ by Mann-Whitney;

c difference between PCOS and Control BMI ≥ 25 kg/m^2^ by Mann-Whitney.

Patients with PCOS reported systematically lower scores in all SF-36 domains (**Table 2**), with statistically significant differences observed in “functional capacity” (PCOS ≠ control BMI < 25 kg/m^2^; *p <* 0.041), “pain” (PCOS ≠ control BMI < 25 kg/m^2^; PCOS ≠ control BMI ≥ 25 kg/m^2^; *p <* 0.0070), “general state of health” (PCOS ≠ control BMI < 25 kg/m^2^; PCOS ≠ control BMI ≥ 25 kg/m^2^; *p <* 0.0001) domains, as well as “vitality” (PCOS ≠ control BMI < 25 kg/m^2^; *p <* 0.0219), “emotional aspects” (PCOS ≠ control BMI < 25 kg/m^2^; *p <* 0.0139), “mental health” (PCOS ≠ control BMI < 25 kg/m^2^; *p <* 0.0141) (**Table 2**). There were no differences between the groups in the areas of “physical aspects” (*p* = 0.2192) and “social aspects” (*p* = 0.1471) (**Table 3**).

**Table 3:** Health-related quality of life - SF-36 according to the study subgroups

	PCOS (n=47)	Control BMI < 25 kg/m^2^(n=47)	Control BM I ≥ 25 kg/m^2^ (n=32)	^*^P-value
Functional capacity	80.0^a^[35.0-100.0]	90.0 [25.0-100.0]	90.0 [45.0-100.0]	0.0041
Physical aspects	75.0 [0.0-100.0]	100.0 [0.0-100.0]	100.0 [0.0-100.0]	0.2192
Pain	51.5^a.c^[10.0-100.0]	74.0 [40.0-100.0]	72.0 [20.0-100.0]	0.0070
Genral satate of health	44.50^a.c^[15.0-66.0]	77.0 [30.0-100.0]	69.5 [32.0-97.0]	<0.0001
Vitality	50.0^a^[10.0-85.0]	60.0 [10.0-90.0]	47.5 [10.0-85.0]	0.0219
Social aspects	68.8 [12.50-100.0]	75.0 [25.0-100.0]	68.8 [25.0-100.0]	0.1471
Emocional aspects	68.8^a^[12.5-100.0]	100.0 [0.0-100.0]	66.7 [00.0-100.0]	0.0139
Mental health	64.0^a^[28.0-80.0]	76.0 [40.0-92.0]	70.0 [32.0-92.0]	0.0141

Values described as median (minimum value - maximum value); QoL = quality of life;

* Kruskal-Wallis; statistical significance = 5%;

a difference between PCOS and control BMI < 25 kg/m^2^ by Mann-Whitney;

b difference between control BMI < 25 kg/m^2^ and control BMI ≥ 25 kg/m^2^ by Mann-Whitney;

c difference between PCOS and control BMI ≥ 25 kg/m^2^ by Mann-Whitney.

## DISCUSSION

This study aimed to evaluate women diagnosed with PCOS, specifically phenotypes A and B, and to compare them with two groups of women without a PCOS diagnosis (one overweight/obese and one of normal BMI) with respect to QoL, HRQoL, and risk of depression. The main findings indicated that all women with PCOS were classified as overweight or obese, although only 47.06% associated their weight gain with PCOS in the initial questionnaire. Additionally, women with PCOS exhibited higher rates of depression risk and reported lower perceived QoL and HRQoL than both control groups.

Obesity is recognized as a major comorbidity associated with PCOS ^(12)^ and plays a crucial role in the disease’s pathophysiology ^(24)^. In this study, patients were randomly recruited during appointments; all were overweight or obese, with no selective bias for obesity. These patients reported poorer perceptions of both QoL and HRQoL, as well as more pronounced signs and symptoms of depression than women in the two control groups without PCOS, regardless of body weight. These results suggest that overweight/obesity alone is likely not the primary driver of adverse outcomes in women with PCOS.

These findings differ from those of Karsten and cols. ^(25)^, who compared women with obesity and a history of infertility (with and without PCOS) and found that in terms of symptoms of anxiety, depression, and QoL perception, poorer outcomes in women with PCOS were more strongly related to obesity than to other PCOS symptoms. Similarly, Himeleinand Thatcher ^(26)^ demonstrated that obesity, along with hirsutism, contributed significantly to reduced QoL, emotional disturbances, and reduced libido. Nevertheless, a 2019 review provides a complementary perspective on the interplay between PCOS and obesity, highlighting that elevated anxiety and depression in women with PCOS hinder the adoption of healthier lifestyles (e.g., regular physical activity, balanced diet), making weight loss - and consequently disease management - more difficult ^(6)^. This bidirectional relationship underscores the complexity of PCOS pathogenesis: obesity increases insulin resistance and hyperinsulinemia, which, in turn, aggravate the reproductive and metabolic dysfunctions typical of PCOS as well as depression ^(10)^.

With respect to depression risk, the current study demonstrated that women with PCOS have a higher risk of depression than women without the disease. This is evidenced by a much greater proportion of women with PCOS at risk of depression (23.5%) compared to the control groups, especially when compared to the BMI-matched group (3.1%) suggesting that depression risk is more closely associated with PCOS-related factors other than obesity. Another study using the Beck Inventory, conducted in Turkey - where the majority of the population is Muslim - concluded that depression was more closely related to infertility, while anxiety was more associated with overweight and obesity ^(27)^. These findings underscore the importance of considering cultural factors in the mental health assessment of women with PCOS.

Perceived QoL, anxiety, and depression risk are widely studied and well-known to be associated with PCOS ^(24,26,28-30)^. The 2023 guideline on PCOS diagnosis and management reiterates previous recommendations that screening for perceived QoL and depression should be conducted at the time of diagnosis using regionally validated instruments; screening should be repeated as needed by clinicians, particularly during specific life stages such as the puerperium. The guideline further recommends referring patients at increased risk for emotional disorders to appropriate treatment ^(12)^.

Regarding HRQoL, as measured by the SF-36 questionnaire, women with PCOS scored worse in domains including functional capacity, pain, general health status, vitality, emotional aspects, and mental health than controls without overweight or obesity (PCOS ≠ control BMI < 25 kg/m^2)^. When compared to overweight/obese controls, women with PCOS also reported significantly worse pain and general health status (PCOS ≠ control BMI ≥ 25 kg/m^2)^. These findings are consistent with prior research comparing women with obesity and infertility, with and without PCOS, which also reported lower emotional domain scores in women diagnosed with PCOS. In contrast, no significant differences were found between groups for physical aspects in that study ^(25)^.

A 2021 study assessed perceived QoL, HRQoL, and anxiety and depression levels in women with PCOS, comparing all four phenotypes. Results showed no significant differences among groups regarding infertility, body weight, and emotional domains; however, phenotypes A and B reported lower HRQoL scores with respect to hirsutism. The study thus recommends evaluating emotional issues in all women with PCOS, regardless of phenotype ^(30)^. These conclusions are supported by a 2013 Endocrine Society position statement, which stated that depression in women with PCOS does not necessarily require the presence of obesity or other disease sequelae, and also recommended assessing depression in all diagnosed patients ^(31)^. It is important to note that this study was conducted at a tertiary referral hospital that treats severe disease cases, including PCOS. Thus, all participants were classified as phenotypes A and B and exhibited all clinical manifestations, limiting comparisons across phenotypes. Moreover, a 2024 study found that infertile women, even without PCOS, require psychological support ^(32)^.

Delayed diagnosis and insufficient information about the disease are additional factors relevant to patient dissatisfaction, as reported in a 2017 study utilizing an online questionnaire with 1,385 women diagnosed with PCOS, aged 18-45, from 32 countries. Findings revealed high dissatisfaction with delayed diagnosis (over two years), consultations with multiple healthcare professionals who failed to identify the syndrome (three or more), and insufficient information on PCOS and its treatment. Importantly, most women from all regions reported not being evaluated for or referred for emotional support ^(1)^. Although we did not evaluate this issue and acknowledges it as a limitation, we also recognize its importance, as prolonged disease duration increases the risk of complications and, consequently, worsens QoL perceptions and depression risk.

Other limitations include the absence of a control group with another chronic disease, which would have allowed assessment of whether having a chronic illness per se is the main factor for low QoL, or whether this is specific to PCOS. Additionally, patients with phenotypes C and D were not included, as the outpatient clinic primarily treats complex disease cases. On the other hand, this study included a substantial sample of women with PCOS and two control groups stratified by body composition, facilitating identification of QoL impairments more closely linked to PCOS. These results reinforce prior evidence, emphasizing the importance of assessing QoL, anxiety, and depression risk in women with PCOS, particularly those with phenotypes A and B, who comprised the focus of this investigation.

In conclusion, the study confirmed previous data showing a higher prevalence of overweight and obesity among women with PCOS phenotypes A and B. However, this factor did not appear to be the primary cause of lower QoL, HRQoL, and increased risk of depression in this group. From a practical perspective, this study reinforces the importance of routine assessment of QoL, HRQoL, and depression risk in women with PCOS during clinical visits. Early identification of emotional and psychological issues can help prevent the onset of severe mental health disorders. The findings also support the need for a multidisciplinary treatment approach involving psychologists, physical educators, nutritionists, and psychiatrists when necessary, alongside endocrinologists and gynecologists, to optimize patient outcomes.

## Data Availability

datasets related to this article will be available upon request to the corresponding author.
